# Diabetes technology in people with diabetes and advanced chronic kidney disease

**DOI:** 10.1007/s00125-024-06244-y

**Published:** 2024-08-08

**Authors:** Rodolfo J. Galindo, Diana Soliman, Daniel Cherñavvsky, Connie M. Rhee

**Affiliations:** 1 University of Miami Miller School of Medicine, Miami, FL USA; 2 University of Virginia Center for Diabetes Technology, Charlottesville, VA USA; 3 David Geffen School of Medicine at UCLA, Los Angeles, CA USA; 4 Veterans Affairs Greater Los Angeles Healthcare System, Los Angeles, CA USA

**Keywords:** Chronic kidney disease, Continuous glucose monitor, Diabetes, Diabetes technology, Review

## Abstract

Diabetes is the leading cause and a common comorbidity of advanced chronic kidney disease. Glycaemic management in this population is challenging and characterised by frequent excursions of hypoglycaemia and hyperglycaemia. Current glucose monitoring tools, such as HbA_1c_, fructosamine and glycated albumin, have biases in this population and provide information only on mean glucose exposure. Revolutionary developments in glucose sensing and insulin delivery technology have occurred in the last decade. Newer factory-calibrated continuous glucose monitors provide real-time glucose data, with predictive alarms, allowing improved assessment of glucose excursions and preventive measures, particularly during and between dialysis sessions. Furthermore, integration of continuous glucose monitors and their predictive alerts with automated insulin delivery systems enables insulin administration to be decreased or stopped proactively, leading to improved glycaemic management and diminishing glycaemic fluctuations. While awaiting regulatory approval, emerging studies, expert real-world experience and clinical guidelines support the use of diabetes technology devices in people with diabetes and advanced chronic kidney disease.

**Graphical Abstract:**

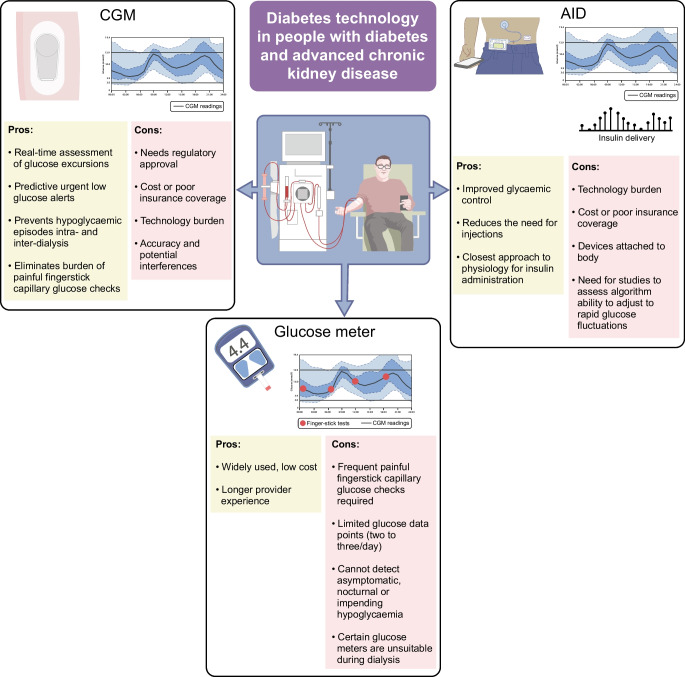

**Supplementary Information:**

The online version contains a slide of the figure for download available at 10.1007/s00125-024-06244-y.

## Introduction

Diabetes is the most common cause of end-stage kidney disease (ESKD), and the most common comorbidity, present in up to 60% of people [1, 2]. Population-based studies show that individuals with ESKD have a disproportionately higher risk of developing severe hypoglycaemic events requiring emergency room visits and/or hospitalisations [3]. These events impose a significant burden of care, are costly to the health system and are associated with a higher risk of permanent sequalae and even death. Moreover, clinicians and individuals may try to avoid hypoglycaemia by allowing liberalisation of glycaemic status at the expense of developing hyperglycaemia. Recent studies using continuous glucose monitoring, which provides a more comprehensive assessment of glucose levels, have demonstrated that dysglycaemic events are very frequent in this population [4–6], with a tendency towards persistent hyperglycaemia. People with diabetes and advanced chronic kidney disease (CKD) also have a high incidence of diabetes-related comorbidities, such as CVD [2], diabetic retinopathy and visual impairment [7] and infections and amputations [8]. Clinicians should aim to optimise glycaemic management while minimising hypoglycaemia, which is paramount for preventing acute glycaemic crises and other diabetes-related complications.

Endogenous insulin clearance is first mediated by the liver and then by the kidneys. The kidneys are responsible for a larger proportion of exogenous insulin metabolism due to its bypass of first-pass metabolism in the liver [1]. Multiple factors in ESKD contribute to hypoglycaemia, including decreased gluconeogenesis, impaired insulin clearance, impaired counterregulatory hormone responses (cortisol), nutritional deficiencies and variable medication effects as a result of haemodialysis (see Text box: ‘Insulin and glucose metabolism in early and advanced CKD’). In addition, the accumulation of ‘uraemic toxins’ is believed to contribute to insulin resistance and postprandial hyperglycaemia [9].


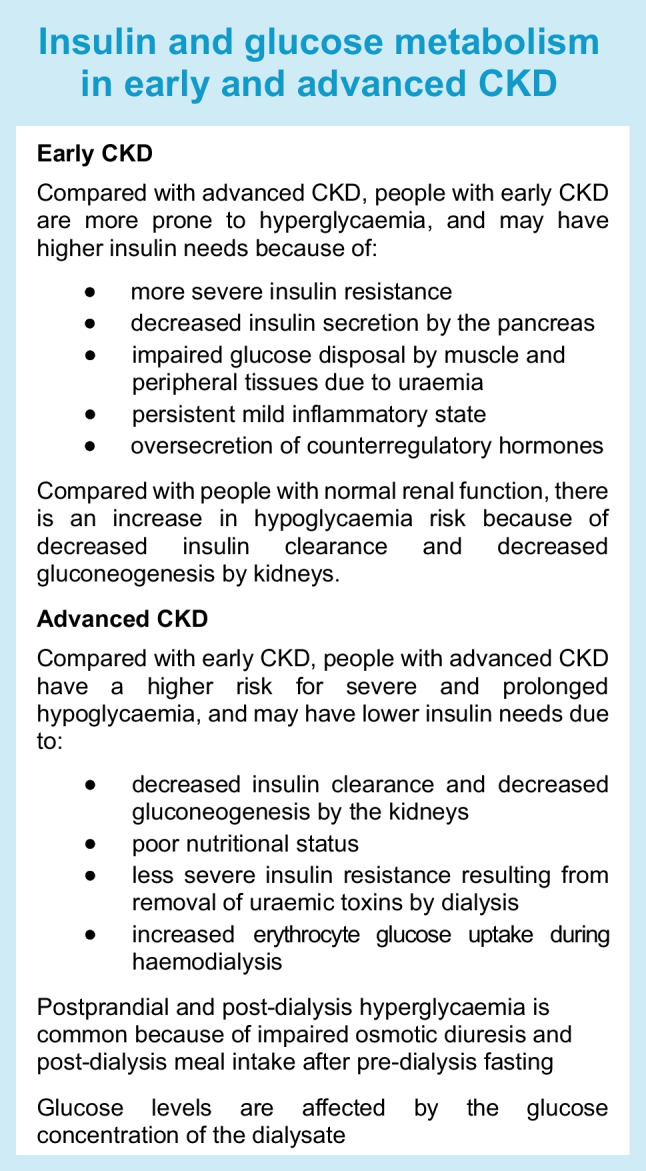


There have been technological advances in diabetes care in the last decade, including the introduction of factory-calibrated continuous glucose monitors (CGMs) and their integration through computerised algorithms to insulin pumps, creating closed-loop systems or automated insulin delivery (AID) systems. However, many of these diabetes technologies have not been tested in validation studies nor approved for use in people with diabetes and ESKD treated with dialysis. In this review, we focus on recent developments in diabetes technology in this population, including glucose meters, CGMs and integrated AID systems.

## Overview of technology developments in glucose sensing

In 1911, Stanley R. Benedict described the first method for assessing glucose levels in humans [10]. This qualitative method measured glucose in the urine and was based on the body’s capacity to reduce free carbonyl groups in glucose using different metals [11]. Since then, numerous scientific advancements have resulted in increasingly precise quantitative methods, culminating in modern techniques that are both highly accurate and relatively simple to use. Currently, three different methods are used for glucose measurement: electrochemical methods (enzymatic and non-enzymatic), optical methods (fluorophore-based and non-flourophore-based) and combinational or other methods (piezoelectric, electromagnetic impedance) [12].

Since the 1970s, enzyme-based electrodes have been widely used for amperometry detection of electrical current differences resulting from the glucose oxidase, hexokinase or glucose dehydrogenase reactions. Older generations of CGMs required people to perform self-monitoring of blood glucose (SMBG) using the finger-stick method, to calibrate the sensor and maintain sensor accuracy. In 2014, the first factory-calibrated CGM was introduced, which removed the need for finger-pricking and SMBG measurements. To estimate glucose levels, a microneedle-based sensor is placed subcutaneously [13, 14], thus creating a minimally invasive method that allows interstitial glucose measurements to be made every 1–5 min that correlate with and have a minimal lag time with blood glucose values [13]. The magnitude of the electrical current corresponds to the concentration of glucose in the tissue at the sensor site and is converted to glucose values through a calibration function. In the newer generation of CGMs, integration of miniaturised sensing electrodes into the lumen of a single hollow microneedle, with an opening facing the dermal space, allowing passive molecular diffusion over a short distance, have minimised the lag time between blood glucose and interstitial fluid glucose concentrations to 4–12 min [13, 15, 16]. These factory-calibrated CGMs contain calibration algorithms, developed by computer engineers, that help correct for sensor drift (gradual loss of accuracy in a CGM sensor's readings over time) by keeping track of the day since insertion and adjusting the calibration function based on the day [13, 15, 16]. Further developments have also been made in electrode materials, enzyme immobilisation techniques and materials used for covering membranes to minimise interference and improve accuracy [14, 17].

The development and refinement of predictive algorithms in factory-calibrated sensors has led to the development of predictive alarms. These alarms alert wearers or integrated devices (e.g. AID systems), providing an opportunity to adjust dietary intake and medications in anticipation of an impending extreme glucose level. A valuable development in this area has been the introduction of predictive low glucose alarms, which alert users or integrated devices of impending hypoglycaemia of ≤3.1 mmol/l (55 mg/dl) within 20 min of an impending hypoglycaemic event, allowing for interventions to prevent hypoglycaemia [18].

Glucose data from a CGM can also be shared wirelessly with a software-based algorithm that further integrates with an insulin infusion pump. Algorithms have been further developed for insulin pumps or insulin delivery systems to calculate more precise insulin doses based on real-time continuous glucose data, creating a closed-loop system. Subsequently, newer functionalities, including suspending insulin before hypoglycaemia occurs or providing small insulin boluses if there is an increasing glucose trend, have also been implemented. Most of these currently available AID systems need users to enter the amount of predicted carbohydrate intake into the pump/algorithm for an insulin bolus dose to be calculated; hence, these are called hybrid closed-loop insulin systems. In addition, some studies have assessed the use of insulin and glucagon together in ‘dual-hormone AID systems’ [e.g. 19]. The colloquially termed ‘artificial pancreas’ is also on the horizon, in which carbohydrate announcement is unnecessary and multiple hormones can be administered [20]. One currently available AID system does not require input of carbohydrate intake, although the user must enter the size of the meal to be consumed [21].

Notwithstanding the clinical impact of these advances, ESKD and/or dialysis treatment may cause abrupt fluid shifts that alter the volume equilibrium between the intravascular and interstitial compartments. Moreover, in the setting of haemodialysis, these changes occur two to four times per week for a few hours during the dialysis treatment sessions. Hence, this can potentially affect the relationship between interstitial glucose sensor readings and actual blood glucose levels. While there is a need for studies to investigate the impact that rapid fluid fluctuations during dialysis may have on interstitial fluid glucose levels, it is expected that machine-learning adjustments can be developed to adapt the algorithms and account for these fluctuations if any; however, to our knowledge this has not been adequately studied to date. Nevertheless, and as shown in previous studies (Tables 1 and 2), the use of continuous glucose monitoring in people with ESKD undergoing haemodialysis provides greater convenience for glycaemic monitoring, may potentially be more accurate than traditional metrics and should be considered for optimal glycaemic management, including avoidance of acute glycaemic complications. In our clinical practice, we have used CGMs and/or AID systems to prevent extreme hyper- and hypoglycaemic events in this population, with vast success. However, it is also important to consider if a sensor needs to be calibrated after rapid volume changes (e.g. during haemodialysis treatment sessions and/or immediately after haemodialysis), using a hybrid model of a CGM and glucose meter system [14].

**Table 1:** Key studies evaluating the accuracy of CGMs in people with CKD and ESKD treated with haemodialysis and/or peritoneal dialysis

Study	Population	CGM duration/device	Accuracy
Matoba, 2020 [51]	T2D, HD (*n*=13)	7 days; FreeStyle Libre Pro (FM), Medtronic iPro2 (CGM), Medisafe Fit (SMBG)	Overall MARD for FM was 18.2% During HD, MARD for FM was 22.8% Percentage of FM readings not in zones A+B was more than four times higher than for CGM
Yajima, 2020 [52]	T2D, HD (*n*=13)	7 days; FreeStyle Libre Pro (FM), Medtronic iPro2 (CGM), OneTouch Ultra Veriovue (SMBG)	MARD for FM was significantly higher than that for CGM (19.5% ± 13.2% vs 8.1% ± 7.6%) FM readings: 49.0% in zone A and 51.0% in zone B CGM readings: 93.3% in zone A and 6.7% in zone B
Hissa, 2021 [53]	T2D, HD (*n*=12)	14 days; Freestyle Libre (CGM), AccuCheck (SMBG)	Overall, MARD 21.4% CEG analyses: 62.5%, 27.1%, 0.0%, 10.4% and 0.0% of readings were in zones A, B, C, D and E, respectively
Mambelli, 2021 [54]	T2D, HD (*n*=20), without diabetes, HD (*n*=11)	14 days; FreeStyle Libre (CGM), Nova Pro Glucose Meter (SMBG)	CEG analyses: 97.6% of all readings were within zones A+B
Ólafsdóttir, 2022 [55]	T1D (*n*=11), T2D (*n*=25), HD (*n*=11), PD (*n*=9)	14 days; Dexcom G5 (CGM), FreeStyle Libre (CGM), HemoCue (SMBG)	MARD (dialysis): Dexcom G5 vs SMBG: 15.5%; Libre vs SMBG 19.3%
Villard, 2022 [56]	T1D, HD (*n*=4), T2D, HD (*n*=12), PTDM, HD (*n*=1)	10 days; Dexcom G6 Pro (CGM), iSTAT System (vBGM) during HD sessions, ContourNext (SMBG) at home	Overall, MARD 13.8–14.3% Parkes error grid analyses: 98.7% and 100% of values were in zones A and B, respectively
Avari, 2023 [57]	T1, HD (*n*=3), T2, HD (*n*=37)	∼4 weeks; Dexcom G6 (CGM), FreeStyle Libre 1 (CGM)	Overall, MARD: Dexcom G6 22.7%; Libre 1 11.3% %15/15 and %20/20 were 29.1% and 45.4% for Dexcom G6 and 73.5% and 85.6% for Libre 1, respectively CEG analyses: 98.9% and 99.8% of readings were in zones A+B for Dexcom G6 and Libre 1, respectively
Horne, 2023 [58]	T1D (*n*=14), T2D (*n*=55), HD	14 days; FreeStyle Libre Pro (FM), personal glucose meter (SMBG)	HD: 97.9% of readings were within zones A+B Non-HD days (based on 225 pairs): 99.1% of readings were within zones A+B
Ng, 2023 [36]	T2D, PD (*n*=30)	14 days; Guardian 3 (CGM), Yellow Springs Instrument glucose analyser (vBGM)	MARD 10.4% No correlations between pH, uraemia, hydration status and MARD 99.9% of readings were in zones A+B
Ling, 2024 [59]	T2D, PD (*n*=30)	8 h; Guardian 3 (CGM)	Overall, MARD 10.4%

CEG, Clark error grid; FM, flash monitoring; HD, haemodialysis; MARD, mean absolute relative difference; PD, peritoneal dialysis; PTMD, post-transplantation diabetes; SMBG, self-monitoring of blood glucose; T1D, type 1 diabetes; T2D, type 2 diabetes; vBGM, venous blood glucose measurement

Zone A represents the area on the error grid where the CGM readings are clinically accurate compared with reference glucose values; zone B represents CGM readings that are still clinically acceptable but may lead to unnecessary treatment or missed treatment opportunities; zone C represents values that would result in overcorrection of acceptable glucose levels; zone D represents ‘clinically dangerous failure to detect and treat’ errors; and zone E is the ‘erroneous treatment’ zone

%15/15 and %20/20 are the percentages of CGM readings that fall within 15% and 20% of the reference glucose values, respectively, when within 0.8 mmol/l (15 mg/dl) or 1.1 mmol/l (20 mg/dl), respectively, of the reference glucose values

**Table 2:** Key studies evaluating glucose trends based on continuous glucose monitoring data in people with CKD and ESKD

Study	Population	CGM duration	Results
Kepenekian, 2014 [60]	T2D, HD (*n*=28)	2.25 days	Reduced HbA_1c_ but not hypoglycaemic events
Gai, 2014 [61]	Diabetes, HD (*n*=12)	6 days	Lower glucose levels during HD, with nadir during the third hour of HD Glycaemic peak occurred 2.5 h after HD
Joubert, 2015 [62]	T1D, T2D, HD (*n*=15)	5 days	Lower mean CGM glucose during HD period Use of CGM improved HbA_1c_ and mean CGM glucose
Vos, 2012 [63]	T2D, CKD but no HD or PD (*n*=25)	2 days	Good correlation of mean CGM glucose with glycated albumin, but poor correlation with HbA_1c_
Mirani, 2010 [64]	T2D, HD (*n*=12)	3 days	High glycaemic variability after HD
Jung, 2010 [65]	T2D, HD (*n*=9)	6 days	Hypoglycaemia during and after HD
Riveline, 2009 [66]	T2D, HD (*n*=19)	4 days	Poor correlation of mean CGM glucose and HbA_1c_
Chantrel, 2014 [67]	T1D, T2D, HD (*n*=33)	3 days	Frequent hypoglycaemia during HD, with higher CGM glucose during early morning the day after HD
Kazempour-Ardebili, 2009 [68]	T2D, HD (*n*=17)	2 days	Frequent hypoglycaemic events, mostly after HD
Sobngwi, 2010 [69]	No diabetes, HD (*n*=14)	2 days (24 h pre HD/HD; 24 h post HD)	Lower glucose during and after HD vs pre-dialysis days, with a nadir during the third hour
Lee, 2013 [38]	T1D, T2D, PD (*n*=25)	3 days	Use of glucose dialysate led to high glucose levels during PD; icodextrin dialysate had no effect on or reduced glucose levels

HD, haemodialysis; PD, peritoneal dialysis; T1D, type 1 diabetes; T2D, type 2 diabetes

## Glycaemic monitoring in ESKD

The ADA and Kidney Disease Improving Global Outcomes (KDIGO) guidelines recommend individualised glycaemic targets in people with diabetes and CKD based on characteristics that could alter the risks and benefits of intensive glycaemic control [8, 22]. A meta-analysis including 83,684 participants found that both high HbA_1c_ levels (≥69.4 mmol/mol [≥8.5%]) and very low HbA_1c_ levels (≤35.5 mmol/mol [≤5.4%]) are associated with an increased mortality risk in people undergoing haemodialysis [8, 23]. In practice, we aim to prevent moderate to severe hyperglycaemia and minimise hypoglycaemia in people with diabetes and ESKD, to avoid acute and chronic complications of diabetes and reduce the risk of death.

### Advantages and disadvantages of glycaemic biomarkers in advanced CKD

HbA_1c_ is the most widely used test for monitoring long-term glycaemic management in people with diabetes. However, in those with ESKD, HbA_1c_ can be falsely low owing to the presence of anaemia, use of erythropoietin-stimulating agents, reduced erythrocyte lifespan from uraemia, erythrocyte lysis during haemodialysis and/or frequent blood transfusions [1]. Fructosamine measurement, which reflects total serum proteins that undergo glycation, can also be inaccurate in ESKD as a result of hypoalbuminaemia, which is a common occurrence in ESKD. Glycated albumin, a ketoamine resulting from the non-enzymatic binding of glucose to albumin, has been proposed as a better indicator of glycaemic management in this population. Glycated albumin is less likely to be affected by confounding factors, although it can also be impacted by low albumin levels, and data suggest it can predict mortality and hospitalisation rates in people with diabetes mellitus and ESKD [8, 24]. However, the lack of availability of glycated albumin in the real-world setting has limited its widespread use. Fructosamine and glycated albumin reflect average blood glucose levels over the previous 2–3 weeks, which is a shorter time frame than is reflected by HbA_1c_. In addition, fructosamine and glycated albumin lack well-established glycaemic targets in populations with ESKD [8, 24]. The advantages and disadvantages of these biomarkers are detailed in Table 3.

**Table 3:** Advantages and disadvantages of glycaemic biomarkers in advanced CKD

Biomarkers	Advantages	Disadvantages
HbA_1c_	• Widely used and implemented • International laboratory standardisation programme • Approved for diagnosis and long-term monitoring	• Low bias in in the presence of reduced erythrocyte lifespan, anaemia, transfusions and use of erythrocyte-stimulating agents and iron therapy • High bias in the presence of metabolic acidosis and elevated urea nitrogen owing to the formation of carbamylated haemoglobin [1] • Overestimation of mean glucose levels in Black individuals [70] • Lower accuracy in advanced CKD [8]
Fructosamine	• Provides intermediate glucose exposure assessment over 2–3 weeks • Not affected by erythrocyte lifespan	• High bias in the presence of hypoalbuminaemia, proteinuria, malnutrition and peritoneal dialysis • Lack of established treatment goals [1, 8]
Glycated albumin	• Provides intermediate glucose exposure assessment over 2–3 weeks • Not affected by erythrocyte lifespan	• Low bias in the presence of hypoalbuminaemia, malnutrition and peritoneal dialysis • Limited availability of testing • Lack of established treatment goals [1, 8]
CGM metrics: • Mean glucose • GMI • TIR (3.9−0 mmol/l) • TBR: ◦ Hypoglycaemia level 1 (<3.9 mmol/l [70 mg/dl]) ◦ Hypoglycaemia level 2 (<3.0 mmol/l [54 mg/dl]) ◦ Number of events ◦ Duration and timing of each event ◦ Prolonged hypoglycaemic events ◦ Nocturnal hypoglycaemic events • TAR: ◦ Hyperglycaemia level 1 (>10 mmol/l [180 mg/dl]) ◦ Hyperglycaemia level 2 (>13.9 mmol/l [250 mg/dl]) ◦ Number of events ◦ Duration and timing of each event	• Eliminates burden of capillary finger-stick testing • Provides real-time feedback after treatment, physical activity and dietary intake • Provides continuous real-time glucose levels during dialysis sessions • Provides insights into glucose trends and patterns over different time periods • Provides predictive low glucose alerts, preventing hypoglycaemic episodes • May reflect more closely actual glucose exposure in the ESKD population [5, 8, 31, 57]	• Needs regulatory approval • More costly and insurance coverage could be limited • Technology burden for some people • Potential for substance interferences or malfunctioning

GMI, glucose management indicator; TAR, time above range; TBR, time below range; TIR, time in range

### Capillary glucose testing

Despite technological advances in blood glucose testing, capillary glucose testing is still widely used for assessing glycaemic management on a daily basis in people with ESKD treated with dialysis. However, beyond concerns specific to people with CKD and ESKD, currently available, US Food and Drug Administration (FDA)-approved, hand-held point-of-care capillary glucose testing meters have additional accuracy and technical limitations, even after post-marketing. A 2017 study of 17 commercially available blood glucose meters demonstrated that accuracy varied substantially, with mean absolute relative differences (MARDs) ranging from 5.6% to 20.8%, with less reliability in participants with anaemia [25], which is a very frequent complication in people with advanced stages of CKD and ESKD. A subsequent study also demonstrated that, among 18 commercially available blood glucose meters, less than half met the accuracy standards [26]. Specifically, among people with ESKD treated with peritoneal dialysis, there are additional concerns about the accuracy of specific blood glucose meters in the setting of icodextrin-based peritoneal dialysis solutions [1]. A previous study reported that icodextrin interference in meters using glucose dehydrogenase pyrroloquinoline quinone methods resulted in falsely high glucose readings and was associated with severe hypoglycaemic events and death [27]. Hence, clinicians should use caution when selecting blood glucose meters for individuals receiving peritoneal dialysis. Clinicians and people with ESKD are also encouraged to check for device safety updates from manufacturers. In addition to accuracy and technical constraints, the main limitation of capillary glucose testing is the infrequent and static information provided on glycaemic excursions compared with CGMs. The expanded use of CGMs has confirmed that glucose management is not a matter of assessing two to three capillary glucose measurements per day, but rather is a continuous and dynamic human process.

### Continuous glucose monitoring

CGMs have the potential to provide many benefits in monitoring and managing diabetes in people with diabetes and ESKD undergoing dialysis and may overcome the limitations of more widely used traditional methods (Table 3). CGMs can provide a more comprehensive evaluation of glycaemic excursions over 24 h and can aid in achieving glycaemic targets in a population in which HbA_1c_ measurement is known to be inaccurate, as well as help to prevent severe dysglycaemic events. In ESKD, the risk of hypoglycaemia is heightened owing to impaired kidney gluconeogenesis, decreased kidney degradation and clearance of insulin, increased erythrocyte glucose uptake during haemodialysis, and nutritional deprivation [1]. A recent study demonstrated that rates of hospitalisation for hyperglycaemia or hypoglycaemia decreased by 18.2% and 17.0%, respectively, in participants with CKD when continuous glucose monitoring was initiated [28]. People with type 2 diabetes and ESKD have been shown to have high levels of glycaemic variability and day-to-day variations in insulin requirements [29]. CGM data reveal that, compared with non-dialysis days, glucose levels are lower during haemodialysis and higher after haemodialysis (Table 2). The ability of CGMs to capture these fluctuations can aid in the adjustment of diet, time of dialysis, exercise, diabetes medications and especially insulin dosing. However, presently, most dialysis clinics check blood glucose levels among haemodialysis recipients before dialysis or less frequently. Hence, point-of-care glucose tests are typically used only if a person manifests symptoms suggestive of hypoglycaemia and many asymptomatic hypoglycaemic episodes may therefore not be detected during haemodialysis. Continuous glucose monitoring has the advantage of monitoring glucose levels before, during and after a haemodialysis session.

Continuous glucose monitoring is recommended for people with diabetes using insulin or at increased risk of hypoglycaemia. With the increased use of CGMs, international consensus groups have developed new glucose metrics, including mean glucose, the glucose management indicator (GMI), mean time in range (TIR; 3.9–10 mmol/l [70–180 mg/dl] glucose), mean time above range (TAR; level1 >10 mmol/l [180 mg/dl]; level 2 >13.9 mmol/l [>250 mg/dl] glucose) and mean time below range (TBR; level 1 <3.9 mmol/l [70 mg/dl]; level 2 <3.0 mmol/l [<54 mg/dl] glucose) [30]. These newer metrics are widely used and implemented in clinical practice; however, many of the goals proposed are based on expert opinion. Based on expert opinion, and given the higher risk of hypoglycaemia, the recommended TIR for individuals with advanced CKD or with ESKD on dialysis is >50%, with a TBR of <1% [30, 31].

Given the inherent bias in HbA_1c_ measurements in ESKD, the KDIGO clinical practice guidelines recommend the use of the GMI, which is derived from CGM data to monitor glycaemic management in individuals whose HbA_1c_ is discordant with directly measured blood glucose levels [8]. The GMI provides a measure of average blood glucose levels and is expressed in units of HbA_1c_ (%) [32]. Some advocate for use of mean glucose levels over the GMI, citing the discordance between the GMI and HbA_1c_ and the confusion it creates for people and providers, but there is a scarcity of studies focused specifically on this issue in ESKD [33, 34]. Regardless, it is expected that measures of average glucose exposure determined by CGMs, such as mean glucose or mean TIR, will overcome the limitations of HbA_1c_ measurement in this group (Table 3). Several studies on the use of diabetes technology in people with CKD and ESKD are ongoing (Table 4).

**Table 4:** Selected ongoing studies assessing diabetes technology in CKD and ESKD

Study and country	Summary/objective and population	Status
Study to determine the efficacy of real-time CGM in preventing hypoglycemia among insulin-treated patients with DM2 on hemodialysis, compared to standard of care (POC BG) (NCT04473430), USA	Randomised controlled crossover trial comparing the use of a Dexcom G6 CGM vs usual care with SMBG with regard to diabetes management outcomes in individuals with T2D on HD.	Status: completed but not yet reported
Continuous glucose monitoring in dialysis patients to overcome dysglycaemia trial (CONDOR) (NCT05509881), USA	RCT investigating whether the use of a Dexcom G6 CGM vs usual care with SMBG (1) enhances glycaemic control (%TIR), (2) reduces hypoglycaemia risk, glycaemic markers (HbA_1c_, glycated albumin, fructosamine) and (3) improves quality of life, diabetes distress and fear of hypoglycaemia in individuals with T1D or T2D on HD (estimated enrolment *n*=122) Intervention: 12 week period of ‘unblinded’ CGM (Dexcom). Control: SMBG at least four times/day. At weeks 6 and 12 of the intervention periods, usual care group will also undergo 10 days of blinded CGM data collection.	Status: recruiting
Dexcom G6 Continuous Glucose Monitoring in Haemodialysis (DEXCOM-HD) (NCT04217161), USA	Pilot study assessing Dexcom G6 accuracy by comparing glucose levels on the CGM device vs blood glucose measurements in adults with T1D or T2D on HD (estimated enrolment *n*=30).	Status: active, not recruiting
Continuous glucose monitoring in patients with diabetes on peritoneal dialysis (NCT06069518), Mexico	Cross-sectional, non-interventional study using a CGM to detect whether the type, dose, route of administration and timing of insulin application are associated with the patterns provided by the CGM (magnitude and duration of periods of hyper- and/or hypoglycaemia) in adults aged >40 years with insulin-dependent T2D on PD. Outcome measures: %TIR, %TBR and %TAR during and after dialysis fluid infusion. Magnitude and duration of periods of hypoglycaemia (<3.9 mmol/l) and hyperglycaemia (>10.0 mmol/l) recorded in 24 h periods. Follow-up: 14 days.	Status: not yet recruiting
Pilot study: FreeStyle Libre Pro Flash continuous glucose monitoring system in subjects with diabetes on haemodialysis (FSL DIAL) (NCT04641650), France	Prospective cohort study assessing continuous glucose monitoring with a FreeStyle Libre Pro in adults with diabetes on HD (estimated enrolment *n*=40). Outcome measures: comparison between estimated HbA_1c_ or GMI using 14 days of CGM data and laboratory HbA_1c_; glucose CV%, %TIR, %TBR and %TAR at sensor removal (day 14).	Status: completed but not yet reported
Blood Sugar Sensing on Maintenance Dialysis (BLOSSOM), USA	Prospective cohort study of Dexcom G6 CGM use in individuals on dialysis (*n*=800).	Status: ongoing, preliminary data (*n*=153) presented at ADA 2023 [71]

CAPD, continuous ambulatory peritoneal dialysis; HD, haemodialysis; MDI, multiple daily injections; PD, peritoneal dialysis; T1D, type 1 diabetes; T2D, type 2 diabetes

For a more comprehensive assessment of glycaemic management, mean glucose, TIR and TBR can be used to define glycaemic targets [30, 31]. It should be noted that even individuals with a low TBR can still have a substantial number of hypoglycaemic events, particularly severe hypoglycaemia. Clinicians should also therefore monitor the frequency of hypoglycaemic events. The target for glycaemic variability metrics, such as %CV, is <36% (%CV = SD of sensor glucose/mean sensor glucose) [30]. This measure assesses glycaemic variability, which should be minimised to avoid glucose fluctuations and hypoglycaemia.

## Use of CGMs in advanced CKD

Some studies have assessed the accuracy of CGMs in the population with advanced CKD; however, most have tested older technology—non-factory-calibrated CGMs—requiring SMBG using finger sticks for calibration, with a shorter time of use (e.g. 7–14 days) and only including one to two dialysis sessions (Table 1). Consequently, the current evidence on the accuracy of CGMs in individuals with advanced CKD derives mostly from sensors that are no longer available or used in clinical practice.

Some more recent studies have assessed the use of newer factory-calibrated CGMs in people treated with haemodialysis or peritoneal dialysis (Tables 1 and 2). Most of these studies have focused on accuracy comparisons, predominantly using capillary glucose testing as the comparator (Table 1), which may not be considered the gold standard by regulatory bodies. People with diabetes and ESKD undergoing dialysis commonly have a high comorbidity burden, frequent hospitalisations, chronic anaemia, difficulty achieving peripheral vein access, low functional status and poor quality of life. Given these unique challenges, it is anticipated that comparative accuracy studies using devices such as the Yellow Springs instruments or Nova Primary Glucose Analyzer System [35], which aim to meet regulatory standards, requiring frequent blood draws and for a prolonged time, will be difficult to perform. More importantly, people with diabetes and ESKD are not benefiting widely from these technological advances because of a lack of regulatory approval, which further widens health disparities. Nevertheless, emerging data on these sensors is encouraging, and helpful for clinical practice.

The literature on newer CGM metrics in people with ESKD is very limited. In a recent study, we compared the relationship between the CGM-derived GMI and laboratory-measured HbA_1c_ in people treated by haemodialysis [4]. HbA_1c_ and GMI (mean ± SD) were 7.1% ± 1.3% (54.1 ± 14.2 mmol/mol) and 7.8% ± 1.1% (61.7 ± 12.0 mmol/mol), respectively (difference 0.74% ± 0.95%). Up to 29% of participants had a discordance between HbA_1c_ and GMI of <0.5%, 22% had a discordance between 0.5% and 1%, and 49% had a discordance of >1%. The GMI had a strong relationship with TIR, but HbA_1c_ underestimated mean glucose levels and the GMI. In accordance with the KDIGO guidelines [8], this suggests that CGM metrics, such as mean glucose, GMI and/or TIR, may be better markers of glycaemic management [4].

While studies using factory-calibrated sensors are emerging, there is a need for research with sensors of longer duration (e.g. 10–14 days), assessed over a period of three to five haemodialysis sessions, as well as research on sensor performance on days when no dialysis sessions are performed. Current studies using older sensor technology demonstrate patterns of lower glucose levels during haemodialysis, reaching the lowest point at the end of dialysis treatment sessions, with a peak in glycaemic levels observed after haemodialysis (Table 2).

There is also a lack of large studies evaluating CGMs in individuals on peritoneal dialysis; however, it has been shown that continuous glucose monitoring can aid in the detection of asymptomatic glucose excursions related to hypertonic exchanges during peritoneal dialysis [36]. In peritoneal dialysis, the glucose concentration of the dialysate, dwell time and status of membrane transport all impact the glycaemic profile [37]. In a small observational study, Lee et al demonstrated that, within 1 h of exchange using glucose-containing dialysate, glucose levels increased [38]. The glycaemic excursion was similar with 1.25% and 2.25% glucose solutions, with more prominent increments observed with 3.86% glucose solutions. Icodextrin solution contains a mixture of glucose polymers that are slowly absorbed and are used as an alternative osmotic agent in peritoneal dialysis [37]. The use of icodextrin dialysate had no effect on, or reduced, glucose levels during peritoneal dialysis [38].

It has been widely established that up to 15–20% of people with diabetes and ESKD transitioning to haemodialysis will experience ‘burnt-out diabetes’. This condition is defined as normalisation of glycaemic levels based on HbA_1c_ <47.5 mmol/mol (6.5%), without the need for medications for at least 6 months [5]. However, one of the major red flags of this definition is the use of HbA_1c_ to determine glycaemic status, given the well-established body of literature demonstrating significant bias in this population. In a recent study using CGMs in individuals with ESKD, investigators from our group demonstrated that people with burnt-out diabetes treated with haemodialysis have higher mean glucose levels, lower TIR and higher TAR (>13.9 mmol/l glucose), and a prolonged duration of TAR (>10 mmol/l; ~4 h per day) compared with people without diabetes treated with haemodialysis [5]. This demonstrates that the term ‘burnt-out diabetes’ may be misleading and further challenges the widely accepted concept of ‘true normalisation of glycaemia’ in this group when determined using a non-reliable test: HbA_1c_.

## Use of AID systems in advanced CKD

AID systems have revolutionised diabetes care, particularly in both children and adults with type 1 diabetes. Extensive evidence supports the benefits of current AID systems in improving glycaemic management, decreasing hypoglycaemia risk and fear of hypoglycaemia, and improving quality of life for people with diabetes. However, the evidence on the use of these devices in people with diabetes and ESKD is still limited.

In a post hoc analysis of an RCT, AID significantly improved glycaemic management in hospitalised participants with type 2 diabetes undergoing haemodialysis (*n*=17) compared with conventional insulin therapy [39]. The closed-loop group had a significantly higher TIR (69.0% in the AID group vs 31.5% in the conventional insulin therapy group) without increasing the risk of hypoglycaemia. The mean CGM glucose level was lower in the AID group (8.1 mmol/l) than in the control group (11.0 mmol/l). Boughton et al conducted a randomised crossover trial in adults with type 2 diabetes requiring dialysis (*n*=26). TIR was significantly higher with AID than with conventional insulin therapy (TIR 52.8% vs 37.7%, respectively) [40], and mean glucose was lower during the closed-loop period (10.1 ± 1.3 mmol/l vs 11.6 ± 2.8 mmol/l; *p*=0.003). There was also a reduction in TBR while using AID. These small studies demonstrate the glycaemic benefit of AID in adults with type 2 diabetes receiving haemodialysis, but further investigations are needed in this population, as well as in the type 1 diabetes population receiving haemodialysis and in individuals receiving peritoneal dialysis.

## Clinical considerations

**Fig. 1:**
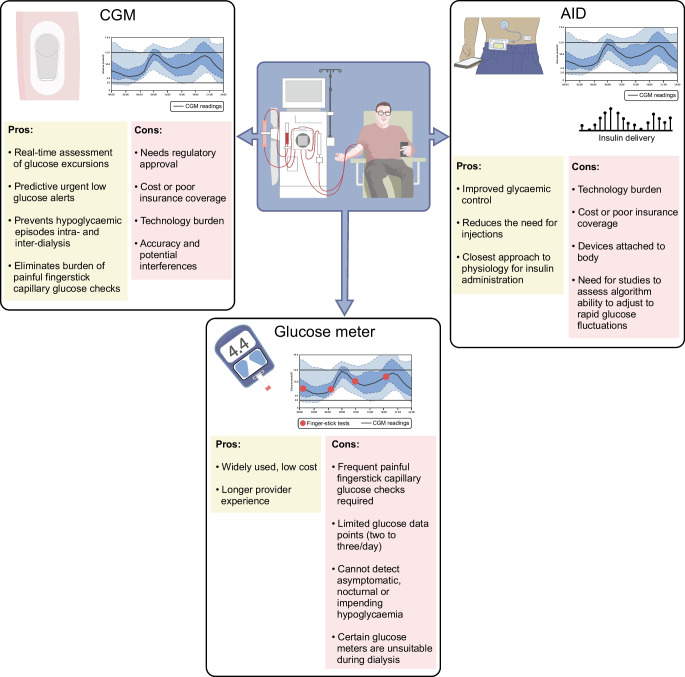
Overview of the advantages and disadvantages of diabetes technology in individuals with diabetes and ESKD. This figure is available as a downloadable slide

In clinical practice, if there is a need to use hypoglycaemic agents in people with diabetes and ESKD, such as insulin and/or sulfonylureas, including in combination with incretin therapy, it is recommended to use a CGM to monitor for glycaemic excursions in real time, especially given the proven benefits of CGMs in preventing hypoglycaemic events with the use of predictive low glucose alerts [18]. Similarly, using AID systems, which integrate the benefits of predictive glucose alerts with decreasing or stopping insulin administration by the insulin pump, is expected to prevent hypoglycaemic events and improve glycaemic management (Fig. 1). Our clinical experience and emerging studies support these recommendations.

CGMs can be a valuable tool for managing diabetes in people with ESKD, but it is important for clinicians to be aware of potential interferences and accuracy considerations. Except for the implantable sensor that uses fluorescence imaging (Eversense), current sensor technology is based on a glucose oxidase reaction. Hence, potential interferences include factors affecting the oxidase reaction, such as uraemic toxins, which can be present at higher concentrations in people with ESKD and have been shown to interfere with CGM readings [41, 42]. Low or high oxygen levels, high altitude, uraemia, icodextrin, low haematocrit and some exogenous substances (maltose, galactose and certain medications) may also pose challenges [37, 43, 44]. Studies have revealed that commonly used substances such as lisinopril, albuterol, acetaminophen, atenolol, ascorbic acid (vitamin C) and red wine, usually at higher than therapeutic doses, can interfere with the readings of electrochemical-based transcutaneous CGMs (those that use a glucose oxidase reaction) [43, 45]. In addition, tetracycline and mannitol have been found to interfere with the fully subcutaneously implantable Eversense glucose sensor [46]. There is also well-established literature reporting a lag time between blood glucose concentrations and interstitial glucose concentrations, which is what CGMs measure. However, predictive algorithms have been developed using glucose data from CGMs to predict the rate and direction of change, including algorithms to predict glucose variability during exercise [47, 48].

In lieu of no evidence in the population with diabetes and ESKD, we usually focus on glucose patterns and use confirmatory capillary glucose checks with validated glucose meters in cases of potential discordance or hypoglycaemic values without symptoms. Studies on interferences are limited and have been performed in non-dialysis populations. Previously, in silico (computer modelling) studies suggested a MARD of 10% for insulin dosing decisions [49]. Real-world experience has shown that a MARD of <15% can still be clinically beneficial for guiding diabetes management, and small differences in MARDs may not translate into clinically relevant changes within certain glucose thresholds [44, 50]. This highlights the importance of considering both ideal and practical accuracy levels when using CGMs in this specific population.

Placement of contemporary CGM sensors (e.g. typically on the arm or abdomen) requires careful consideration in the ESKD population and should be avoided in the ipsilateral (same) arm when arteriovenous fistulas (AVFs) and/or arteriovenous grafts (AVGs) are present. In general, any blood draws, infusions and/or blood pressure measurements are contraindicated in the AVF/AVG arm because of the risk of infection, clotting and/or damage to arteriovenous access, which is the ‘lifeline’ for individuals receiving dialysis, and this cautionary approach should also be extended to CGM sensors. Hence, subcutaneous sensors that are inserted just under the skin may be preferred in people with ESKD if placed in the non-AVF/AVG arm. It should also be noted that people with advanced CKD have a higher rate of coagulopathy and bleeding (e.g. due to uraemic platelet dysfunction), which may impact sensor functioning after insertion. Hence, providing training in the proper placement of CGMs is of paramount importance in this population. Compared with older sensors, newer factory-calibrated sensors are smaller, more user-friendly and less invasive, and lower the burden of diabetes care by not requiring frequent finger-prick glucose calibrations. They are also more accessible, although there are still cost constraints.

## Future directions for diabetes technology in ESKD

While AID systems can effectively learn glucose patterns and insulin needs and deliver insulin based on current trends in people without ESKD, there is a need for validation studies of current AID algorithms in the population with ESKD. An ideal AID system for those with ESKD would learn individual patterns, including insulin clearance rates and sensitivity changes during and after haemodialysis or peritoneal dialysis. There is a need for more studies using CGM data in this cohort to enable personalised algorithms to be developed using machine learning. Table 4 provides details of selected ongoing studies that are assessing the use of diabetes technology in CKD and ESKD. It should also be underscored that ESKD and diabetes are conditions that disproportionately affect people with impaired social determinants of health and/or from racial and ethnic minority groups; hence, there is a compelling need to improve access to diabetes technologies in these vulnerable populations and to optimise dissemination of the benefits of CGMs to populations with broad health literacy.

## Conclusion

Despite significant advances in diabetes technology and therapeutics, achieving optimal glycaemic management in people with diabetes and ESKD remains a challenge. CGMs and AID systems, although awaiting regulatory approval for people on dialysis, have already proved beneficial to both individuals with advanced CKD and clinicians in real-world settings (Fig. 1). They provide valuable insights into an individual’s glucose patterns and allow for more personalised treatment plans, preventing impending and asymptomatic hypoglycaemic and/or hyperglycaemic episodes, especially during dialysis sessions. CGM data can assist providers in tailoring insulin and diabetes medications and lifestyle changes for people with ESKD. We anticipate that machine-learning modelling using CGM data to predict glucose trends and fluctuations will reduce the impact of inter- and intra-dialysis variations on sensor performance. AID systems that can adjust target blood glucose levels based on the dialysis modality (in-centre haemodialysis, peritoneal dialysis and home haemodialysis) could significantly improve glycaemic management. We encourage clinicians, researchers and manufacturers to advocate for and pursue additional research and regulatory approval of these newer devices in this population to decrease disparities.

## Supplementary Information

Below is the link to the electronic supplementary material.

Figure slide (PPTX 152 KB)

## Abbreviations

AID: Automated insulin delivery
AVF: Arteriovenous fistula
AVG: Arteriovenous graft
CGM: Continuous glucose monitor
CKD: Chronic kidney disease
ESKD: End-stage kidney disease
GMI: Glucose management indicator
KDIGO: Kidney Disease Improving Global Outcomes
MARD: Mean absolute relative difference
SMBG: Self-monitoring of blood glucose
TAR: Time above range
TBR: Time below range
TIR: Time in range
