# Correction: Diabetes technology in people with diabetes and advanced chronic kidney disease

**DOI:** 10.1007/s00125-024-06278-2

**Published:** 2024-09-10

**Authors:** Rodolfo J. Galindo, Diana Soliman, Daniel Cherñavvsky, Connie M. Rhee

**Affiliations:** 1 University of Miami Miller School of Medicine, Miami, FL USA; 2 University of Virginia Center for Diabetes Technology, Charlottesville, VA USA; 3 David Geffen School of Medicine at UCLA, Los Angeles, CA USA; 4 Veterans Affairs Greater Los Angeles Healthcare System, Los Angeles, CA USA

**Correction: Diabetologia**

10.1007/s00125-024-06244-y

Unfortunately, the name Daniel Cherñavvsky was spelt incorrectly in the author list. The original article has been corrected.

