# Supplementary material for: Diabetes technology in people with diabetes and advanced chronic kidney disease
Source: Diabetologia. 2024 Aug 8;67(10):2129–42. doi: 10.1007/s00125-024-06244-y (PMC11446991; doi:10.1007/s00125-024-06244-y)
Supplement: Supplementary file 1 — Figure slide (PPTX 152 KB) [file 125_2024_6244_MOESM1_ESM.pptx]

## Slide 1
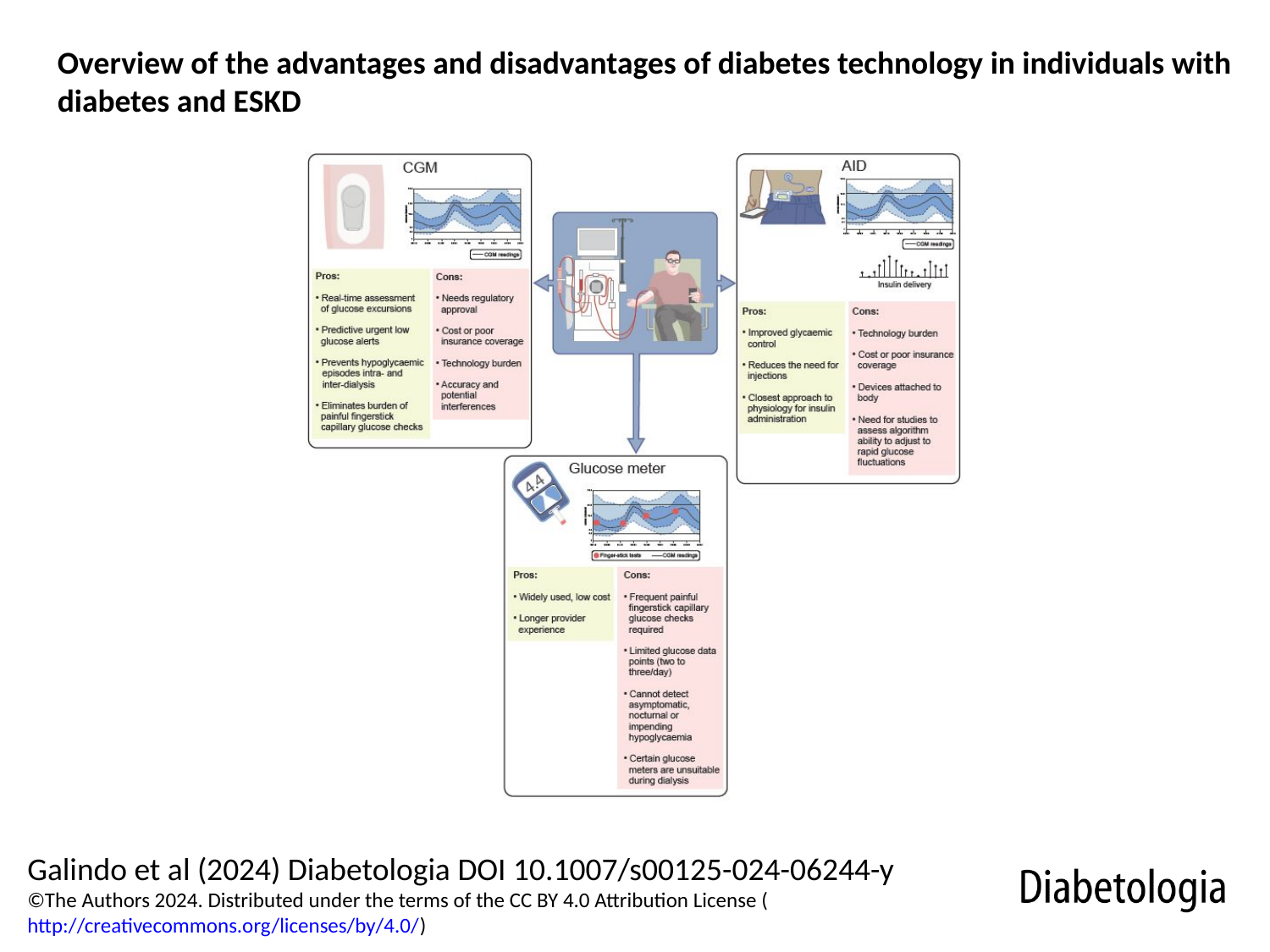

Overview of the advantages and disadvantages of diabetes technology in individuals with diabetes and ESKD
Galindo et al (2024) Diabetologia DOI 10.1007/s00125-024-06244-y
©The Authors 2024. Distributed under the terms of the CC BY 4.0 Attribution License (http://creativecommons.org/licenses/by/4.0/)
